# Supplementary material for: Beta RBD boost broadens antibody-mediated protection against SARS-CoV-2 variants in animal models
Source: Cell Rep Med. 2021 Oct 23;2(11):100450. doi: 10.1016/j.xcrm.2021.100450 (PMC8536561; doi:10.1016/j.xcrm.2021.100450)
Supplement: Document S1. Figures S1–S5 [file mmc1.pdf]

Cell Reports Medicine, Volume *2*

Supplemental information

Beta RBD boost broadens antibody-mediated protection against SARS-CoV-2 variants in animal models

Daniel J. Sheward, Marco Mandolesi, Egon Urgard, Changil Kim, Leo Hanke, Laura Perez Vidakovics, Alec Pankow, Natalie L. Smith, Xaquin Castro Dopico, Gerald M. McInerney, Jonathan M. Coquet, Gunilla B. Karlsson Hedestam, and Ben Murrell

Supplementary Figures

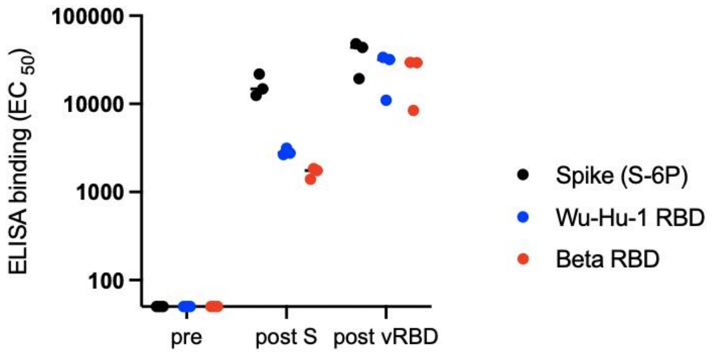

Supplementary figure 1. ELISA spike and RBD binding titers. Binding antibody responses by ELISA are shown for samples prior to immunization (pre) and 2 weeks following the second spike immunization (post S) or variant RBD boost (post vRBD). Binding was assessed against Spike ectodomain with 6 Proline mutations stabilizing the pre-fusion conformation (S-6P, black), as well as against RBD matching Wu-Hu-1 (blue) and the Beta VOC (red). Related to Figure 2.

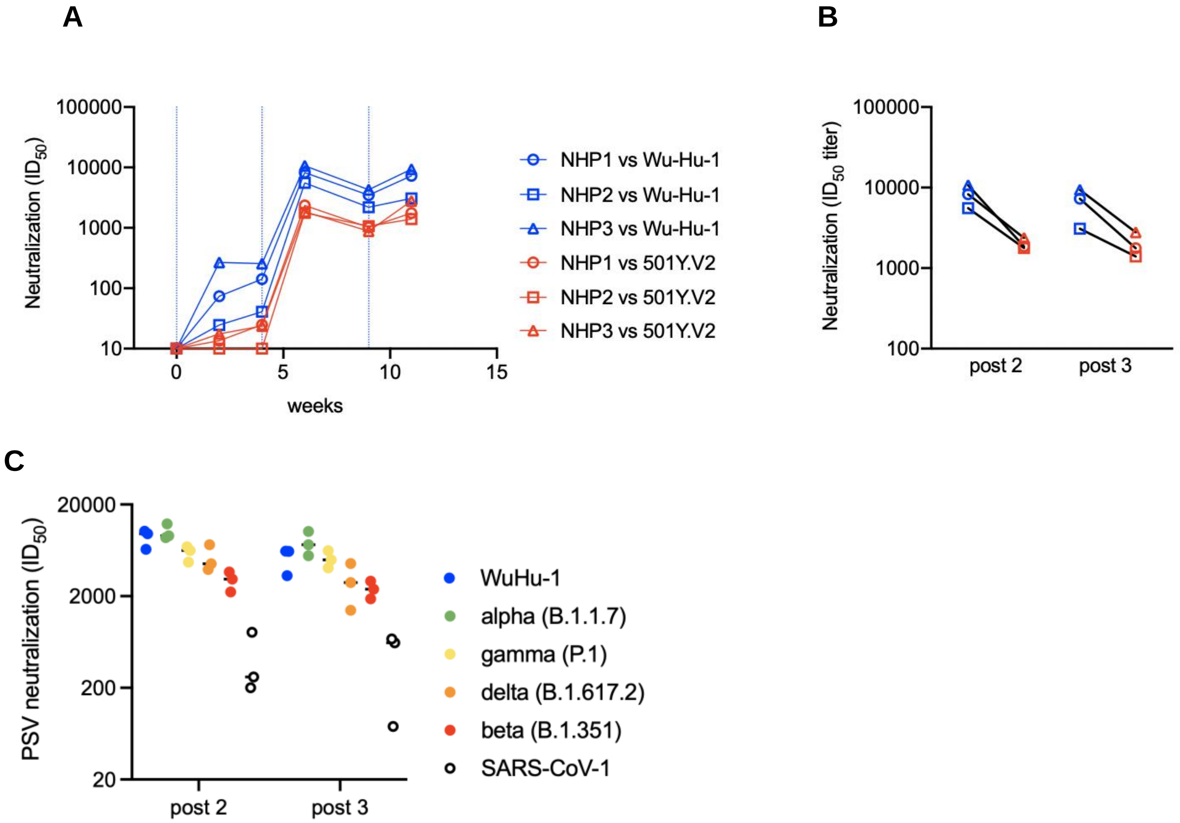

Supplementary figure 2. (**a**) Longitudinal neutralizing antibody responses against Wu-Hu-1 (blue) and 501Y.V2 (red) for plasma samples from [21], where three rhesus macaques (NHP1-NHP3) were immunized with three doses of Wu-Hu-1 spike (100 µg) in Matrix-M™ adjuvant. Vertical blue lines indicate the timing of immunizations (at 0, 4, and 9 weeks). (**b-c**) Comparison of the neutralizing antibody titers at 6 weeks (post 2) and 11 weeks (post 3) illustrating that reduced titers to beta (red) and other VoCs (**c**) compared to Wu-Hu-1 (blue) persisted after a third homotypic spike boost. Related to Figure 2.

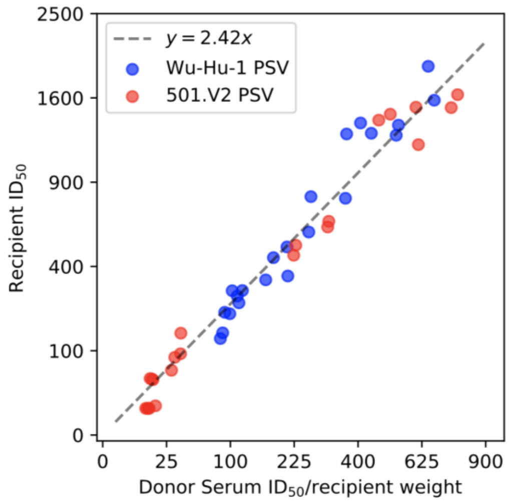

Supplementary figure 3: Relationship between donor and recipient titers following passive immunization. Mice were passively immunized with 200 ul of plasma, resulting in titers approximately 10-fold lower than in the donor. Correcting for weight, Spearman's r = 0.98 (after a variance-stabilizing square-root transform). Related to Figure 3.

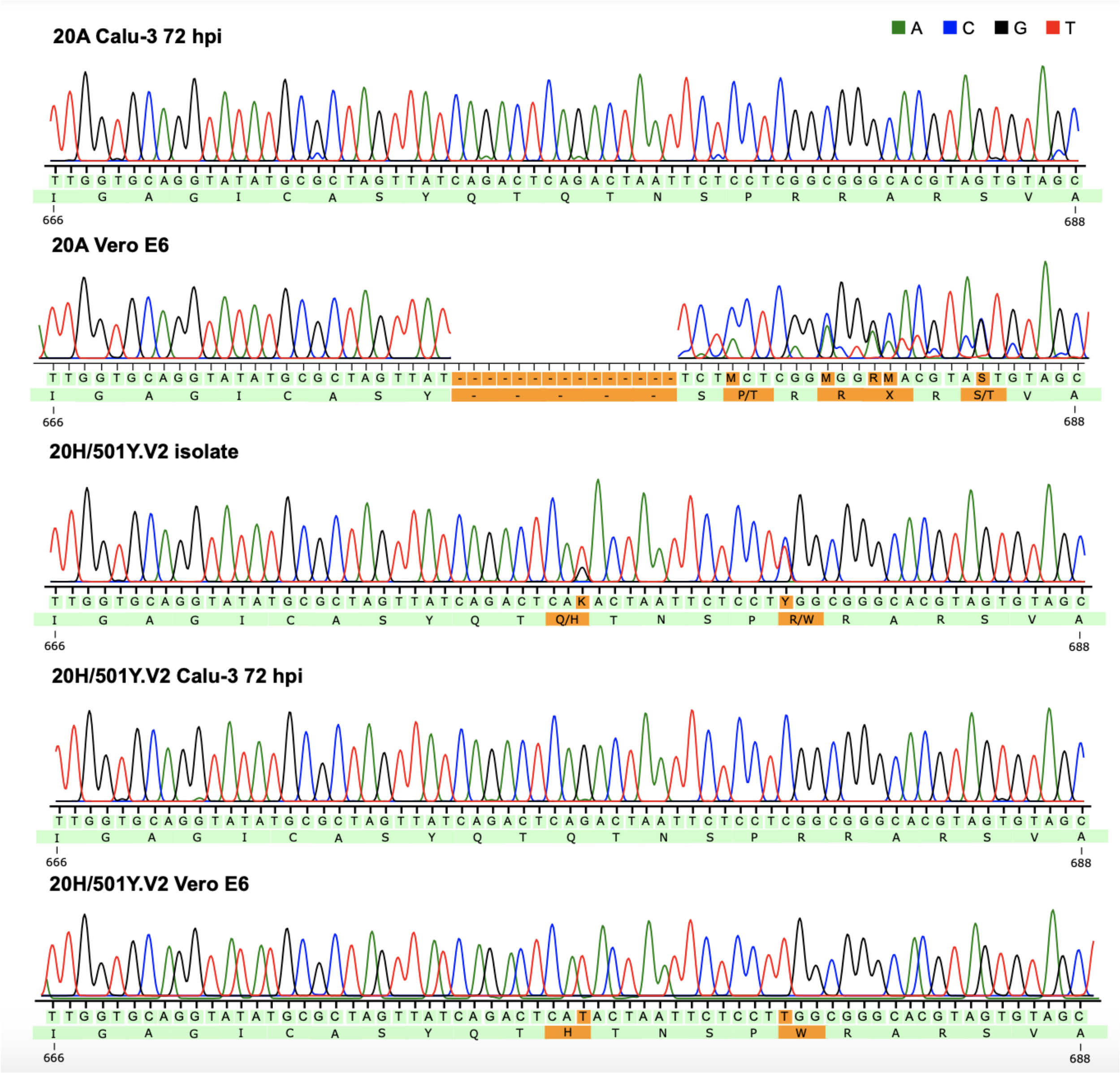

Supplementary figure 4. Expansion of SARS-CoV-2 isolates in Vero E6 but not Calu-3 cells rapidly selected for mutations and deletions proximal to the furin cleavage site. Challenge stocks used in this study were produced in Calu-3 cells, and confirmed by Sanger sequencing to harbour no high frequency cell culture adaptation mutations in spike. Electropherograms spanning the furin cleavage site from sanger sequencing of amplified viral RNA are shown for virus cultured in Vero E6 or Calu-3 cells demonstrating the rapid loss of the furin recognition sequence upon culture in Vero E6 cells but not Calu-3 cells. Received stock of 501Y.V2 ("20H/501Y.V2 isolate") had a mixture of intact/knocked-out furin site. Related to Figure 3.

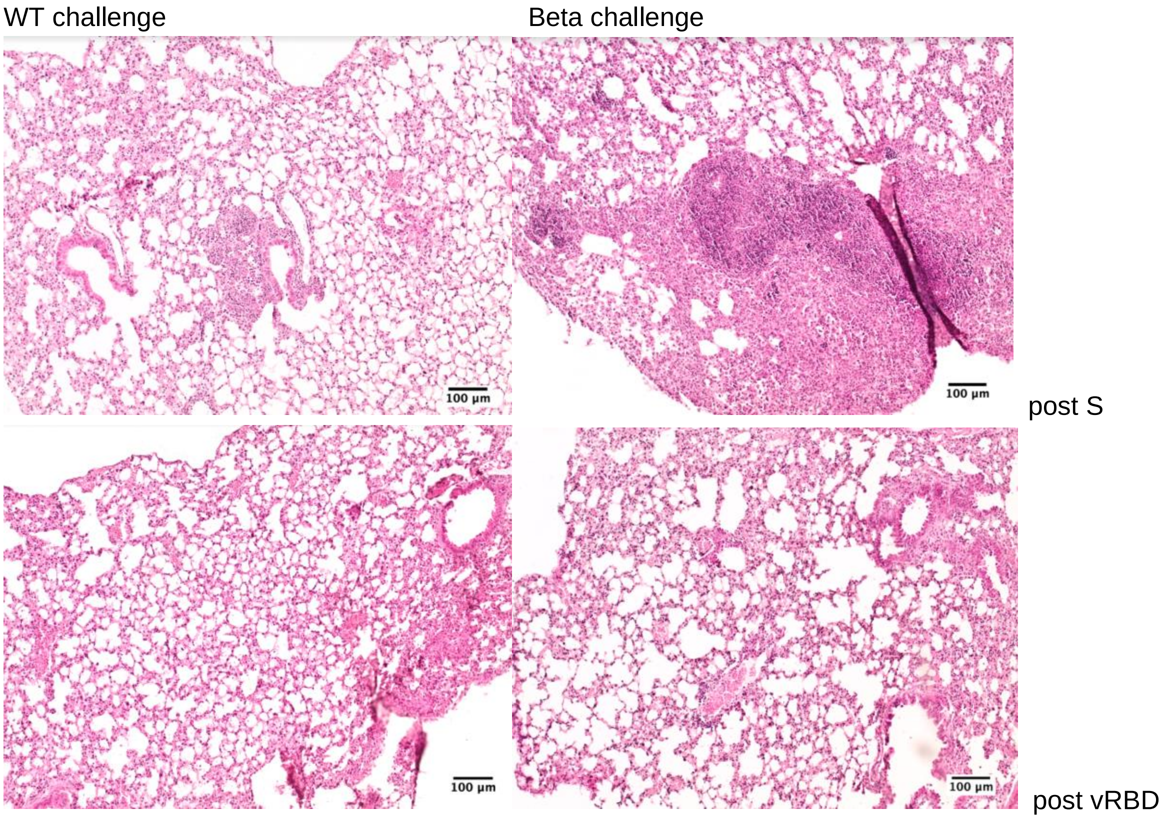

Supplementary figure 5. Lung histopathology in surviving mice. Representative images of Hematoxylin and Eosin stained lung sections from surviving mice at day 14, illustrating typical histopathology in each group. Scale bar represents 100 µm. Related to Figure 3f.
