# Supplementary material for: Beta RBD boost broadens antibody-mediated protection against SARS-CoV-2 variants in animal models
Source: Cell Rep Med. 2021 Oct 23;2(11):100450. doi: 10.1016/j.xcrm.2021.100450 (PMC8536561; doi:10.1016/j.xcrm.2021.100450)
Supplement: Document S2. Article plus supplemental information [file mmc2.pdf]

Cell Reports
Medicine

Article

Beta RBD boost broadens antibody-mediated protection against SARS-CoV-2 variants in animal models

Graphical abstract

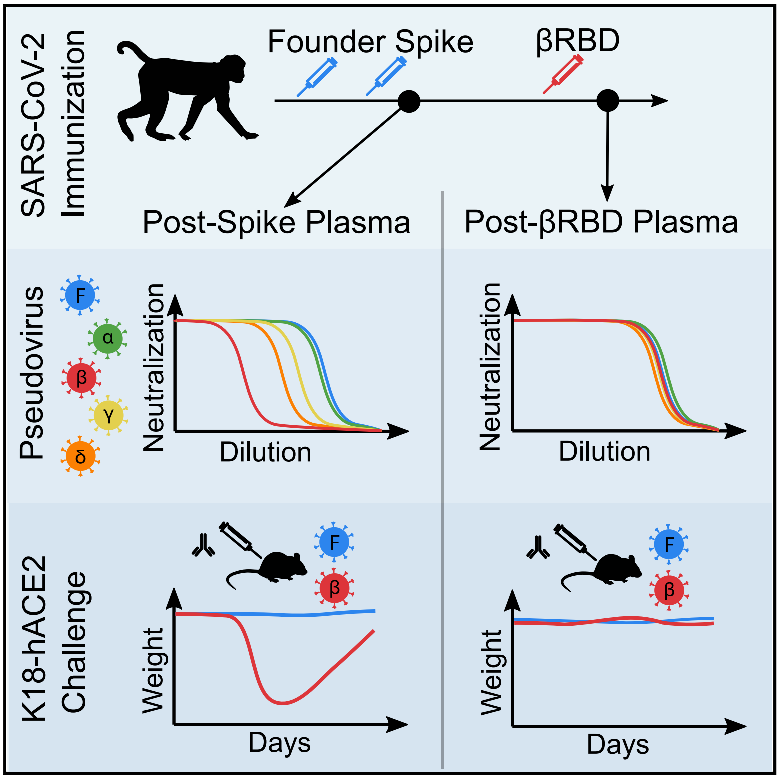

Authors

Daniel J. Sheward, Marco Mandolesi, Egon Urgard, ..., Jonathan M. Coquet, Gunilla B. Karlsson Hedestam, Ben Murrell

Correspondence

daniel.sheward@ki.se (D.J.S.),
gunilla.karlsson.hedestam@ki.se (G.B.K.H.),
benjamin.murrell@ki.se (B.M.)

In brief

The emergence and spread of antibody-resistant SARS-CoV-2 variants of concern (VOCs) threatens to diminish vaccine efficacy. Sheward et al. show, in rhesus macaques and K18-hACE2 mice, that reduced vaccine protection against VOCs can be restored by broadening antibody responses with a third, heterotypic RBD booster immunization.

Highlights

- Heterotypic RBD boost elicits cross-neutralizing antibody responses in macaques
- No evidence that original antigenic sin hinders booster immunizations with beta RBD
- Pre-boost plasma only partially protects K18-hACE2 mice from beta variant challenge
- Post-boost plasma affords full protection from beta variant challenge

Sheward et al., 2021, Cell Reports Medicine 2, 100450
November 16, 2021 © 2021 The Authors.
https://doi.org/10.1016/j.xcrm.2021.100450

CellPress

Cell Reports Medicine

CellPress
OPEN ACCESS

Article

Beta RBD boost broadens antibody-mediated protection against SARS-CoV-2 variants in animal models

Daniel J. Sheward,[1,2,3,*] Marco Mandolesi,[1,3] Egon Urgard,[1,3] Changil Kim,[1] Leo Hanke,[1] Laura Perez Vidakovics,[1] Alec Pankow,[1] Natalie L. Smith,[1] Xaquin Castro Dopico,[1] Gerald M. McInerney,[1] Jonathan M. Coquet,[1,4] Gunilla B. Karlsson Hedestam,[1,4,*] and Ben Murrell[1,4,5,*]

[1]Department of Microbiology, Tumor and Cell Biology, Karolinska Institutet, Stockholm, Sweden

[2]Division of Medical Virology, Institute of Infectious Diseases and Molecular Medicine, Faculty of Health Sciences, University of Cape Town, Cape Town, South Africa

[3]These authors contributed equally

[4]These authors contributed equally

[5]Lead contact

*Correspondence: daniel.sheward@ki.se (D.J.S.), gunilla.karlsson.hedestam@ki.se (G.B.K.H.), benjamin.murrell@ki.se (B.M.)

https://doi.org/10.1016/j.xcrm.2021.100450

SUMMARY

Severe acute respiratory syndrome-coronavirus 2 (SARS-CoV-2) variants of concern (VOCs) with resistance to neutralizing antibodies are threatening to undermine vaccine efficacy. Vaccination and infection have led to widespread humoral immunity against the pandemic founder (Wu-Hu-1). Against this background, it is critical to assess the outcomes of subsequent immunization with variant antigens. It is not yet clear whether heterotypic boosts would be compromised by original antigenic sin, where pre-existing responses to a prior variant dampen responses to a new one, or whether the memory B cell repertoire would bridge the gap between Wu-Hu-1 and VOCs. We show, in macaques immunized with Wu-Hu-1 spike, that a single dose of adjuvanted beta variant receptor binding domain (RBD) protein broadens neutralizing antibody responses to heterologous VOCs. Passive transfer of plasma sampled after Wu-Hu-1 spike immunization only partially protects K18-hACE2 mice from lethal challenge with a beta variant isolate, whereas plasma sampled following heterotypic RBD boost protects completely against disease.

INTRODUCTION

At least 27 candidate severe acute respiratory syndrome-coronavirus 2 (SARS-CoV-2) vaccines have already entered phase 3 clinical trials. A number of these demonstrated high efficacy,[1–5] significantly reducing morbidity and mortality, and are being rolled out globally. This first generation of vaccines all encode or deliver a spike glycoprotein derived from the pandemic founder strain, Wu-Hu-1.[6]

Driven by multiple evolutionary forces,[7] SARS-CoV-2 is evading immune responses and threatening to undermine current prevention and mitigation strategies. Globally, novel variants of concern (VOCs) are increasingly dominating the pandemic (Figure 1). Of particular concern is the surge of variants harboring spike mutations that confer resistance to prior immunity, such as 501Y.V2 (B.1.351, "beta").[8–11] This underpins the substantially reduced vaccine efficacies observed in trials in South Africa, where this variant was circulating at high frequency.[12,13] Recently, significant numbers of vaccine breakthrough infections have been observed during infection waves dominated by the delta (B.1.617.2) variant, which also displays reduced sensitivity to neutralization.[14–16] Updated vaccines are likely required to protect against current and future mutated variants. Importantly, by the time these are rolled out, a significant proportion of the global population are likely to be seropositive from either infection or immunization with Wu-Hu-1-based vaccines. A relevant question now is whether a single additional dose will be sufficient to induce robust neutralizing antibody responses to VOCs in seropositive individuals and whether these boosts are sufficient to confer protection. Importantly, the first exposure to a pathogen can shape future responses to mutated variants. This immunological imprinting or original antigenic sin[17] is well described for influenza A virus, where protection is highest against the first strain encountered and diminished against those encountered later in life.[18,19] It is crucial for the design of updated vaccines and regimens to determine whether existing immunity dampens antibody responses to new VOCs or whether a heterotypic boost can efficiently recruit cross-protective memory responses.

RESULTS

To address this, we immunized three rhesus macaques with two doses of soluble prefusion-stabilized Wu-Hu-1 spike protein (2 μg), adjuvanted with saponin-based Matrix-M (Novavax AB, Uppsala, Sweden), with a 1-month interval between doses, mimicking an immunization schedule for approved SARS-CoV-2 vaccines. After a single dose, neutralizing antibodies were

This is an open access article under the CC BY license (http://creativecommons.org/licenses/by/4.0/).

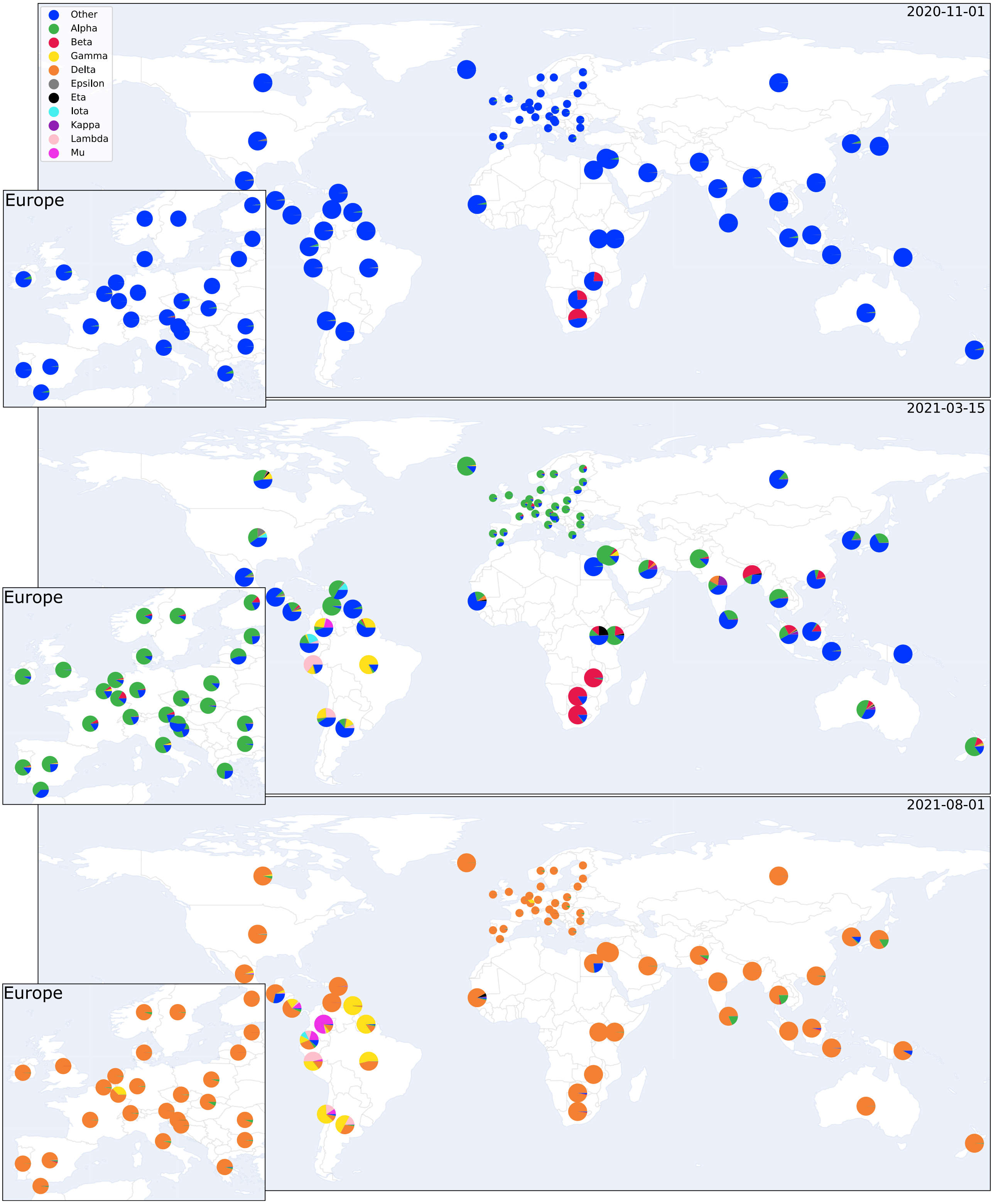

Figure 1. SARS-CoV-2 variants can rapidly come to dominate the global genomic landscape

The global distribution and estimated country-level proportions of deposited SARS-CoV-2 genomes for eight variants, shown for 1 November 2020 (top), 4.5 months later for 15 March 2021 (middle), and as of 1 August 2021 (bottom). Proportions over time are estimated from GISAID[20] genome metadata, using a locally weighted multinomial regression model (see STAR Methods).

CellPress
OPEN ACCESS

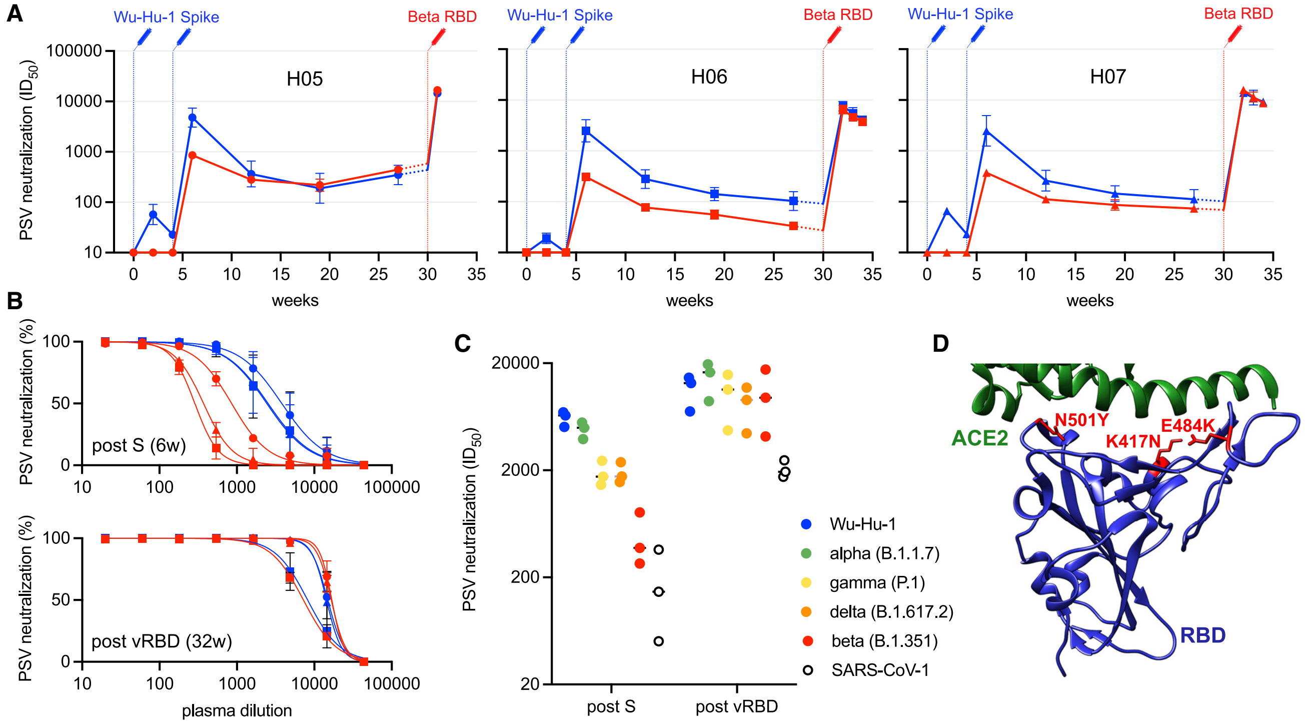

Figure 2. Heterotypic RBD boost drives a potent cross-neutralizing antibody response

(A) Neutralizing antibody responses over time to Wu-Hu-1 (blue) and beta (red) pseudotyped viruses (PSVs) are shown for three immunized macaques: H05; H06; and H07. Syringes indicate the timing of immunizations (blue: Wu-Hu-1 spike at 0 and 4 weeks; red: beta RBD at 30 weeks). Titers from 27 to 30 weeks (shown with dashed lines) have been extrapolated for clarity. Error bars depict the geometric SD from at least 3 technical repeats.

(B) Although PSV neutralization (reduction in infectivity, as a %) of beta was significantly reduced at 6 weeks, corresponding to peak responses 2 weeks following the second spike dose (post-S), neutralization was restored following subsequent heterotypic RBD boost (post-vRBD) such that beta (red) and Wu-Hu-1 (blue) were potently neutralized at similar titers in all three animals. Error bars depict the SD from at least 3 technical repeats.

(C) Heterotypic RBD boost also restored the reduced neutralizing antibody titers against VOCs alpha (green), gamma (yellow), and delta (orange) as well as improved the neutralization of the more distantly related SARS-CoV-1 (open circles).

(D) Depiction of the RBD immunogen (PDB: 6MOJ),[22] used as a heterotypic boost in this study, that incorporates the three RBD mutations (located in red) defining lineage 20H/501Y.V2 (beta). The cellular receptor, ACE2, is shown in green.

detectable against Wu-Hu-1, but not against the beta variant (Figure 2). Neutralizing antibody responses against Wu-Hu-1 were substantially boosted by the second immunization (“post-S”), with a peak geometric mean titer (GMT) of 3,980, and then waned over the following months (Figure 2), as also reported in immunized humans.[21] Notably, the beta VOC was on average 9-fold (range: 5.6- to 12.2-fold) less potently neutralized (GMT = 451 at peak), consistent with the responses observed in humans following vaccination.[9–11]

6 months after their first immunization, macaques were boosted with either 2 μg (H05), 10 μg (H06), or 50 μg (H07) of soluble 501Y.V2 RBD in 50 μg Matrix-M adjuvant. One macaque (H05) was terminated 5 days after immunization, due to an unrelated illness that had begun prior to the third immunization, and was sampled for detailed follow-up studies of antibody specificities. The two other macaques (H06 and H07) were followed for 4 weeks. In all three animals, beta RBD efficiently boosted responses (Figure S1) that potently cross-neutralized both Wu-Hu-1 and beta, with similar titers (Figures 2A–2C; Wu-Hu-1 GMT = 11,795; beta GMT = 12,595). This suggests that the heterotypic boost may have preferentially recruited cross-neutralizing memory B cells. Indeed, heterotypic boost improved neutralization of other VOCs, including delta, alpha, and gamma, as well as the more distantly related SARS-CoV-1 (Figure 2C). In contrast, for macaques previously immunized with three doses of Wu-Hu-1 spike,[23] the reduced neutralization of beta (and other VOCs) compared to Wu-Hu-1 remained after the third homotypic spike immunization (Figure S2).

To determine whether recovery of neutralizing antibody titers to beta afforded a biologically relevant improvement in protective immunity, mice transgenic for human ACE2 (K18-hACE2)[24] were passively immunized intraperitoneally (i.p.) with plasma samples taken either 2 weeks following the second spike immunization (n = 8) (post-S) or 1 to 2 weeks following the RBD booster immunization (n = 8; “post-vRBD”). Passive immunization conferred titers approximately 10-fold lower than donor plasma (Figure S3), and macaque polyclonal antibodies were not rapidly cleared following xenotransfusion, with an unchallenged mouse still maintaining titers >1,400 after 5 days (data not shown). Mice were then challenged intranasally with 2.4 × 10^6 RNA copies of either beta or “wild-type” (WT) (encoding a spike matching Wu-Hu-1) virus (corresponding to 100 plaque-forming units [PFUs]

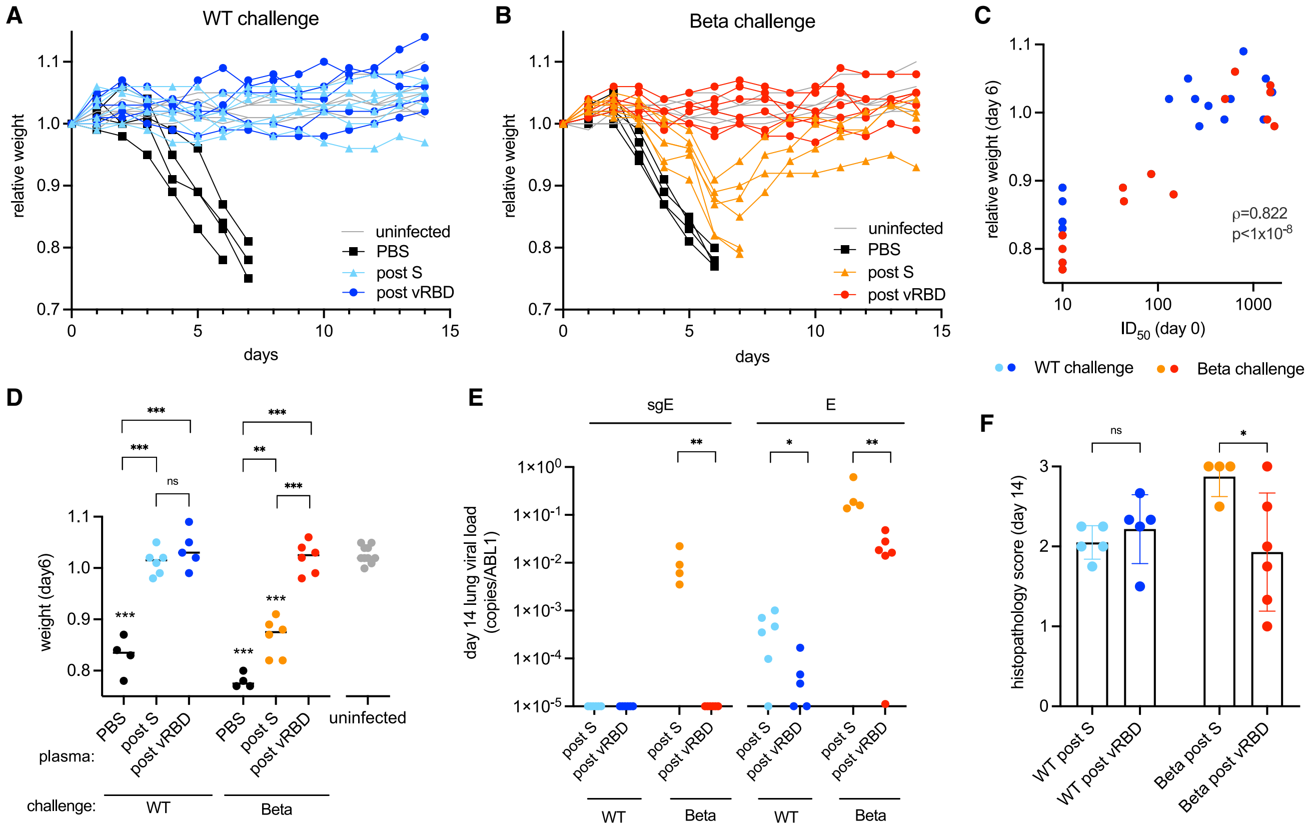

Figure 3. Heterotypic RBD boost restores protection against 501Y.V2 in passively immunized k18-hACE2 mice

(A and B) Weight loss following challenge with either (A) "wild-type" ("WT") or (B) 501Y.V2 virus for K18-hACE2 mice passively immunized with macaque plasma sampled post-S or post-vRBD. Control mice mock immunized with PBS and subsequently challenged ("PBS") are shown in black, and uninfected littermates housed in the same cages ("uninfected") are shown in gray.

(C) Pseudovirus-neutralizing antibody titers against the challenge spike (infective dose 50 [ID_{50}]) in passively immunized mice on the day of challenge are associated with infection and disease severity summarized as weight loss 6 days following challenge. Titers below the limit of detection of the assay (20) are plotted as 10.

(D) Weight loss at day 6 for each group. Unchallenged littermates housed in the same cages (gray) are shown; PBS, mock immunized mice (black). Post-S, passive immunization with plasma following the second spike immunization (6-week plasma); post-vRBD, passive immunization with plasma from macaques boosted with variant (beta) RBD (31- or 32-week plasma). Groups displaying significant weight loss compared to uninfected mice are annotated above the points for that group.

(E) Viral loads in lung tissue on day 14 quantified as the ratio of the copies of either viral genomic envelope (E) or subgenomic transcripts (sgE) to the number of copies of housekeeping gene ABL1. Undetectable copies are plotted on the baseline (1×10^{-5}).

(F) Pulmonary pathology scores in H&E-stained lung sections (89 slides in total, with a mean of 3.3 slides per mouse; range: 2–5). All statistical comparisons are summarized as $^{**}p < 0.01$ and $^{***}p < 0.001$; ns, not significant. Error bars depict SDs.

of beta or 86 PFUs of WT) and monitored for weight loss, a reliable proxy for disease severity (Figure 3).[25]

Across all groups, protection was strongly correlated with the neutralizing antibody titers to the challenge virus on the day of challenge (Spearman's $\rho = 0.822$; $p < 1 \times 10^{-8}$; Figure 3C). All control mice that did not receive plasma (PBS only) succumbed to disease when challenged with either variant, showing precipitous weight loss starting around 3 days post-challenge (Figures 3A, 3B, and 3D). Passive transfer of post S plasma conferred complete protection against weight loss following infection with WT virus (Figures 3A and 3D), but not from beta (Figures 3B and 3D), clearly demonstrating that evasion of the antibody response by this VOC was sufficient to cause disease. Notably, passive transfer of post-vRBD plasma protected against both WT and beta (Figures 3A–3D). Analysis of lung viral loads at day 14 post-infection demonstrated heightened viral clearance in mice transfused with post-vRBD plasma, in particular, in mice infected with beta (Figure 3E). In addition, hematoxylin and eosin staining was performed on lungs 14 days post-infection to assess the pathological effects of virus infection. WT and beta virus both induced signs of alveoli septa thickening and infiltration of inflammatory cells, although mice infected with beta also demonstrated a loss in general tissue architecture (Figures S5 and 3F). Even among only the surviving mice, those transfused with post-vRBD plasma displayed significantly reduced pathological scores following beta infection than did mice that received post-S plasma (Figure 3F).

DISCUSSION

For many licensed SARS-CoV-2 vaccines, reduced efficacy has been observed against the beta variant.[12,26,27] Moreover, the decay of vaccine-elicited antibody titers[21,28] suggests that, over time, protection will wane further. Consistent with reduced vaccine efficacy against beta, we show that breakthrough infection and disease occur in K18-hACE2 mice passively immunized with plasma from rhesus macaques immunized with two doses of adjuvanted WT spike protein.

Cross-species passive immunization and challenge experiments have been used to show antibody-mediated protection conferred by influenza vaccination (human to mouse)[29] and, more recently, to characterize protection from WT SARS-CoV-2 (macaque to hamster).[30] Our data further demonstrate the utility of this approach for investigating protection against SARS-CoV-2 variants.

The ability of vaccines to broaden existing responses to new variants is still largely unclear. Despite weak immunogenicity of soluble, monomeric RBD as a priming antigen,[23] heterotypic RBD administered as a boost elicited a potent recall response in non-human primates. This was robust to the boosting dose, and effective as low as 2 μg, possibly aided by a dose-sparing effect of Matrix-M.[31] Although reduced neutralization of beta was evident following 2 doses of Wu-Hu-1 spike, both Wu-Hu-1 and beta were potently neutralized following heterotypic (beta) RBD boost. In animal challenge models, neutralizing antibodies following passive immunization represented a robust correlate of protection such that the restoration of neutralizing antibody titers to beta also translated into protective immunity.

The potent, cross-neutralizing antibody response that arises following a heterotypic boost indicates that original antigenic sin does not represent a significant barrier to the acquisition of protective immunity against current SARS-CoV-2 VOCs. This is largely consistent with recently reported results from beta spike encoding mRNA (mRNA1273.351, Moderna) booster vaccinations.[32,33] The observation that immunization with RBD (and not whole spike) was capable of inducing robust neutralizing antibody responses is particularly promising as RBD is a small, stable protein that can be rapidly synthesized and efficiently expressed. Here, in the immunized animal sampled only 5 days post-vRBD boost, neutralizing titers (against both Wu-Hu-1 and beta) were already elevated, suggesting these titers are the product of a rapidly activated population of antibody-secreting cells. Further, this time course indicates that successive rounds of affinity maturation likely were not required for neutralization of beta but rather that vRBD-specific antibody responses could be boosted from the pool of existing cross-neutralizing memory B cells primed by Wu-Hu-1. This is consistent with the neutralization of multiple VOCs (including alpha, gamma, and delta) with comparable potency. Cross-neutralization of delta is particularly important, given the high transmissibility and global prevalence of this variant.

Taken together, these data indicate that potent, cross-neutralizing, and cross-protective antibody responses can be recruited with heterotypic SARS-CoV-2 immunogens following a primary exposure and identify soluble RBD booster immunizations as an attractive strategy to broaden vaccine protection from new SARS-CoV-2 variants.

Limitations of the study

Although we show that a heterotypic RBD immunization can efficiently boost protective cross-neutralizing antibody responses, these results are based on only a small number of non-human primates, and we did not characterize the development and breadth of individual antibody lineages. Furthermore, we did not have a well-controlled, homotypic boost arm for comparison. The animals in the previous study that received three doses of WT spike were immunized with a substantially higher dose and received the third dose significantly sooner, preventing a direct comparison. Thus, although a heterotypic RBD boost elicits potent cross-neutralizing responses, further work is required to quantify the benefits of this approach over a third homotypic spike (or RBD) boost.

STAR★METHODS

Detailed methods are provided in the online version of this paper and include the following:

- KEY RESOURCES TABLE
- RESOURCE AVAILABILITY
  - Lead contact
  - Materials availability
  - Data and code availability
- EXPERIMENTAL MODEL AND SUBJECT DETAILS
  - Ethics statement
  - Animal models
  - Cell lines
- METHOD DETAILS
  - Immunizations
  - Pseudotyped virus neutralization assays
  - Virus production and quantification
  - K18-hACE2 mice challenge
- QUANTIFICATION AND STATISTICAL ANALYSIS
  - Non-linear Multinomial Regression for VOC frequency estimation
  - ID_{50} titers
  - Histology
  - Statistical Analysis

SUPPLEMENTAL INFORMATION

Supplemental information can be found online at https://doi.org/10.1016/j.xcrm.2021.100450.

ACKNOWLEDGMENTS

We thank Dr. Bengt Eriksson and all personnel at Astrid Fagraeus laboratory for expert assistance with rhesus macaques. We thank Monika Àdori for assistance processing samples. We also thank Novavax, AB, Uppsala, Sweden, for generously making the Matrix-M adjuvant available. We gratefully thank James Voss, Deli Huang, and Jesse Bloom for reagents. We gratefully acknowledge Penny Moore and the NICD (South Africa) for providing the B.1.351 (501Y.V2) spike plasmid, which was generated using funding from the South African Medical Research Council. We gratefully acknowledge the G2P-UK National Virology consortium funded by MRC/UKRI (grant ref. MR/W005611/1) and

the Barclay Lab at Imperial College for providing the B.1.617.2 spike plasmid. pCDNA3.3_CoV2_B.1.1.7 and pCDNA3.3_CoV2_P.1 were gifts from David Nemazee. We thank Jonas Klingström for sharing the Swedish SARS-CoV-2 isolate and Alex Sigal from the Africa Health Research Institute for providing the 501Y.V2 isolate. For SARS-CoV-2 variant data from GISAID, we gratefully acknowledge all data contributors, i.e., the authors and their originating laboratories responsible for obtaining the specimens and their submitting laboratories that generated the genetic sequence and metadata and shared via the GISAID Initiative the data on which the variant frequency estimates in this research are based. This project has received funding from the European Union's Horizon 2020 research and innovation programme under grant agreement no. 101003653 (CoroNAb) to G.M.M., G.B.K.H., and B.M.; from the Swedish Research Council to G.M.M., J.M.C., G.B.K.H., and B.M.; and from Karolinska Institutet Development Office and Karolinska Institutet President's Fund to G.M.M., G.B.K.H., and B.M. E.U. is supported by a Wenner Gren Fellowship.

AUTHOR CONTRIBUTIONS

Conceptualization, D.J.S., M.M., E.U., J.M.C., G.B.K.H., and B.M.; formal analysis, D.J.S. and B.M.; funding acquisition, G.M.M., J.M.C., G.B.K.H., and B.M.; investigation, D.J.S., M.M., E.U., C.K., A.P., N.L.S., and X.C.D.; methodology, D.J.S., M.M., E.U., A.P., N.L.S., J.M.C., G.B.K.H., and B.M.; resources, C.K., L.H., and L.P.V.; software, B.M.; supervision, L.H., G.M.M., J.M.C., G.B.K.H., and B.M.; visualization, D.J.S., A.P., and B.M.; writing – original draft, D.J.S. and B.M.; writing – review & editing, all authors.

DECLARATION OF INTERESTS

The authors declare no competing interests.

Received: May 11, 2021
Revised: August 20, 2021
Accepted: October 18, 2021
Published: October 23, 2021

REFERENCES

1. Baden, L.R., El Sahly, H.M., Essink, B., Kotloff, K., Frey, S., Novak, R., Diemert, D., Spector, S.A., Rouphael, N., Creech, C.B., et al.; COVE Study Group (2021). Efficacy and safety of the mRNA-1273 SARS-CoV-2 vaccine. N. Engl. J. Med. *384*, 403–416.
2. Polack, F.P., Thomas, S.J., Kitchin, N., Absalon, J., Gurtman, A., Lockhart, S., Perez, J.L., Pérez Marc, G., Moreira, E.D., Zerbini, C., et al.; C4591001 Clinical Trial Group (2020). Safety and efficacy of the BNT162b2 mRNA Covid-19 vaccine. N. Engl. J. Med. *383*, 2603–2615.
3. Voysey, M., Clemens, S.A.C., Madhi, S.A., Weckx, L.Y., Folegatti, P.M., Aley, P.K., Angus, B., Baillie, V.L., Barnabas, S.L., Bhorat, Q.E., et al.; Oxford COVID Vaccine Trial Group (2021). Safety and efficacy of the ChAdOx1 nCoV-19 vaccine (AZD1222) against SARS-CoV-2: an interim analysis of four randomised controlled trials in Brazil, South Africa, and the UK. Lancet *397*, 99–111.
4. Keech, C., Albert, G., Cho, I., Robertson, A., Reed, P., Neal, S., Plested, J.S., Zhu, M., Cloney-Clark, S., Zhou, H., et al. (2020). Phase 1-2 trial of a SARS-CoV-2 recombinant spike protein nanoparticle vaccine. N. Engl. J. Med. *383*, 2320–2332.
5. Logunov, D.Y., Dolzhikova, I.V., Shcheblyakov, D.V., Tukhvatulin, A.I., Zubkova, O.V., Dzharullaeva, A.S., Kovyrshina, A.V., Lubenets, N.L., Grousova, D.M., Erokhova, A.S., et al.; Gam-COVID-Vac Vaccine Trial Group (2021). Safety and efficacy of an rAd26 and rAd5 vector-based heterologous prime-boost COVID-19 vaccine: an interim analysis of a randomised controlled phase 3 trial in Russia. Lancet *397*, 671–681.
6. Wu, F., Zhao, S., Yu, B., Chen, Y.-M., Wang, W., Song, Z.-G., Hu, Y., Tao, Z.-W., Tian, J.-H., Pei, Y.-Y., et al. (2020). A new coronavirus associated with human respiratory disease in China. Nature *579*, 265–269.
7. Martin, D.P., Weaver, S., Tegally, H., San, E.J., Shank, S.D., Wilkinson, E., Giandhari, J., Naidoo, S., Pillay, Y., Singh, L., et al. (2021). The emergence and ongoing convergent evolution of the N501Y lineages coincides with a major global shift in the SARS-CoV-2 selective landscape. medRxiv. https://doi.org/10.1101/2021.02.23.21252268.
8. Tegally, H., Wilkinson, E., Giovanetti, M., Iranzadeh, A., Fonseca, V., Giandhari, J., Doolabh, D., Pillay, S., San, E.J., Msomi, N., et al. (2020). Emergence and rapid spread of a new severe acute respiratory syndrome-related coronavirus 2 (SARS-CoV-2) lineage with multiple spike mutations in South Africa. bioRxiv. https://doi.org/10.1101/2020.12.21.20248640.
9. Wang, P., Nair, M.S., Liu, L., Iketani, S., Luo, Y., Guo, Y., Wang, M., Yu, J., Zhang, B., Kwong, P.D., et al. (2021). Antibody resistance of SARS-CoV-2 variants B.1.351 and B.1.1.7. Nature *593*, 130–135.
10. Zhou, D., Dejnirattisai, W., Supasa, P., Liu, C., Mentzer, A.J., Ginn, H.M., Zhao, Y., Duyvesteyn, H.M.E., Tuekprakhon, A., Nutalai, R., et al. (2021). Evidence of escape of SARS-CoV-2 variant B.1.351 from natural and vaccine-induced sera. Cell *184*, 2348–2361.e6.
11. Wu, K., Werner, A.P., Moliva, J.I., Koch, M., Choi, A., Stewart-Jones, G.B.E., Bennett, H., Boyoglu-Barnum, S., Shi, W., Graham, B.S., et al. (2021). mRNA-1273 vaccine induces neutralizing antibodies against spike mutants from global SARS-CoV-2 variants. bioRxiv. https://doi.org/10.1101/2021.01.25.427948.
12. Madhi, S.A., Baillie, V., Cutland, C.L., Voysey, M., Koen, A.L., Fairlie, L., Padayachee, S.D., Dheda, K., Barnabas, S.L., Bhorat, Q.E., et al.; NGS-SA Group; Wits-VIDA COVID Group (2021). Efficacy of the ChAdOx1 nCoV-19 Covid-19 vaccine against the B.1.351 variant. N. Engl. J. Med. *384*, 1885–1898.
13. Shinde, V., Bhikha, S., Hoosain, Z., Archary, M., Bhorat, Q., Fairlie, L., Lalloo, U., Masilela, M.S.L., Moodley, D., Hanley, S., et al. (2021). Preliminary efficacy of the NVX-CoV2373 Covid-19 vaccine against the B.1.351 variant. medRxiv. https://doi.org/10.1101/2021.02.25.21252477.
14. Elliott, P., Haw, D., Wang, H., Eales, O., Walters, C.E., Ainslie, K.E.C., Atchison, C., Fronterre, C., Diggle, P.J., Page, A.J., et al. (2021). REACT-1 round 13 final report: exponential growth, high prevalence of SARS-CoV-2 and vaccine effectiveness associated with Delta variant in England during May to July 2021. medRxiv. https://doi.org/10.1101/2021.09.02.21262979.
15. Planas, D., Veyer, D., Baidaliuk, A., Staropoli, I., Guivel-Benhassine, F., Rajah, M.M., Planchais, C., Porrot, F., Robillard, N., Puech, J., et al. (2021). Reduced sensitivity of SARS-CoV-2 variant Delta to antibody neutralization. Nature *596*, 276–280.
16. Davis, C., Logan, N., Tyson, G., Orton, R., Harvey, W., Haughney, J., Perkins, J., Peacock, T.P., Barclay, W.S., Cherepanov, P., et al. (2021). Reduced neutralisation of the Delta (B.1.617.2) SARS-CoV-2 variant of concern following vaccination. bioRxiv. https://doi.org/10.1101/2021.06.23.21259327.
17. Francis, T. (1960). On the doctrine of original antigenic sin. Proc. Am. Philos. Soc. *104*, 572–578.
18. Lessler, J., Riley, S., Read, J.M., Wang, S., Zhu, H., Smith, G.J.D., Guan, Y., Jiang, C.Q., and Cummings, D.A.T. (2012). Evidence for antigenic seniority in influenza A (H3N2) antibody responses in southern China. PLoS Pathog. *8*, e1002802.
19. Gostic, K.M., Ambrose, M., Worobey, M., and Lloyd-Smith, J.O. (2016). Potent protection against H5N1 and H7N9 influenza via childhood hemagglutinin imprinting. Science *354*, 722–726.
20. Shu, Y., and McCauley, J. (2017). GISAID: global initiative on sharing all influenza data - from vision to reality. Euro Surveill. *22*, 30494.
21. Widge, A.T., Rouphael, N.G., Jackson, L.A., Anderson, E.J., Roberts, P.C., Makhene, M., Chappell, J.D., Denison, M.R., Stevens, L.J., Pruijssers, A.J., et al.; mRNA-1273 Study Group (2021). Durability of responses after SARS-CoV-2 mRNA-1273 vaccination. N. Engl. J. Med. *384*, 80–82.

22. Lan, J., Ge, J., Yu, J., Shan, S., Zhou, H., Fan, S., Zhang, Q., Shi, X., Wang, Q., Zhang, L., and Wang, X. (2020). Structure of the SARS-CoV-2 spike receptor-binding domain bound to the ACE2 receptor. Nature *581*, 215–220.

23. Mandolesi, M., Sheward, D.J., Hanke, L., Ma, J., Pushparaj, P., Perez Vidakovics, L., Kim, C., Àdori, M., Lenart, K., Loré, K., et al. (2021). SARS-CoV-2 protein subunit vaccination of mice and rhesus macaques elicits potent and durable neutralizing antibody responses. Cell Rep. Med. *2*, 100252.

24. McCray, P.B., Jr., Pewe, L., Wohlford-Lenane, C., Hickey, M., Manzel, L., Shi, L., Netland, J., Jia, H.P., Halabi, C., Sigmund, C.D., et al. (2007). Lethal infection of K18-hACE2 mice infected with severe acute respiratory syndrome coronavirus. J. Virol. *81*, 813–821.

25. Winkler, E.S., Bailey, A.L., Kafai, N.M., Nair, S., McCune, B.T., Yu, J., Fox, J.M., Chen, R.E., Earnest, J.T., Keeler, S.P., et al. (2020). SARS-CoV-2 infection of human ACE2-transgenic mice causes severe lung inflammation and impaired function. Nat. Immunol. *21*, 1327–1335.

26. Shinde, V., Bhikha, S., Hoosain, Z., Archary, M., Bhorat, Q., Fairlie, L., Lalloo, U., Masilela, M.S.L., Moodley, D., Hanley, S., et al.; 2019nCoV-501 Study Group (2021). Efficacy of NVX-CoV2373 Covid-19 vaccine against the B.1.351 variant. N. Engl. J. Med. *384*, 1899–1909.

27. Abu-Raddad, L.J., Chemaitelly, H., and Butt, A.A.; National Study Group for COVID-19 Vaccination (2021). Effectiveness of the BNT162b2 Covid-19 vaccine against the B.1.1.7 and B.1.351 variants. N. Engl. J. Med. *385*, 187–189.

28. Doria-Rose, N., Suthar, M.S., Makowski, M., O'Connell, S., McDermott, A.B., Flach, B., Ledgerwood, J.E., Mascola, J.R., Graham, B.S., Lin, B.C., et al.; mRNA-1273 Study Group (2021). Antibody persistence through 6 months after the second dose of mRNA-1273 vaccine for Covid-19. N. Engl. J. Med. *384*, 2259–2261.

29. Nachbagauer, R., Feser, J., Naficy, A., Bernstein, D.I., Guptill, J., Walter, E.B., Berlanda-Scorza, F., Stadlbauer, D., Wilson, P.C., Aydillo, T., et al. (2021). A chimeric hemagglutinin-based universal influenza virus vaccine approach induces broad and long-lasting immunity in a randomized, placebo-controlled phase I trial. Nat. Med. *27*, 106–114.

30. Corbett, K.S., Nason, M.C., Flach, B., Gagne, M., O' Connell, S., Johnston, T.S., Shah, S.N., Edara, V.V., Floyd, K., Lai, L., et al. (2021). Immune correlates of protection by mRNA-1273 immunization against SARS-CoV-2 infection in nonhuman primates. bioRxiv. https://doi.org/10.1101/2021.04.20.440647.

31. Tian, J.-H., Patel, N., Haupt, R., Zhou, H., Weston, S., Hammond, H., Logue, J., Portnoff, A.D., Norton, J., Guebre-Xabier, M., et al. (2021). SARS-CoV-2 spike glycoprotein vaccine candidate NVX-CoV2373 immunogenicity in baboons and protection in mice. Nat. Commun. *12*, 372.

32. Wu, K., Choi, A., Koch, M., Elbashir, S., Ma, L., Lee, D., Woods, A., Henry, C., Palandjian, C., Hill, A., et al. (2021). Variant SARS-CoV-2 mRNA vaccines confer broad neutralization as primary or booster series in mice. bioRxiv. https://doi.org/10.1101/2021.04.13.439482.

33. Wu, K., Choi, A., Koch, M., Ma, L., Hill, A., Nunna, N., Huang, W., Oestreicher, J., Colpitts, T., Bennett, H., et al. (2021). Preliminary analysis of safety and immunogenicity of a SARS-CoV-2 variant vaccine booster. medRxiv. https://doi.org/10.1101/2021.05.05.21256716.

34. Cele, S., Gazy, I., Jackson, L., Hwa, S.-H., Tegally, H., Lustig, G., Giandhari, J., Pillay, S., Wilkinson, E., Naidoo, Y., et al.; Network for Genomic Surveillance in South Africa; COMMIT-KZN Team (2021). Escape of SARS-CoV-2 501Y.V2 from neutralization by convalescent plasma. Nature *593*, 142–146.

35. Hanke, L., Vidakovics Perez, L., Sheward, D.J., Das, H., Schulte, T., Moliner-Morro, A., Corcoran, M., Achour, A., Karlsson Hedestam, G.B., Hällberg, B.M., et al. (2020). An alpaca nanobody neutralizes SARS-CoV-2 by blocking receptor interaction. Nat. Commun. *11*, 4420.

36. Hsieh, C.-L., Goldsmith, J.A., Schaub, J.M., DiVenere, A.M., Kuo, H.-C., Javanmardi, K., Le, K.C., Wrapp, D., Lee, A.G., Liu, Y., et al. (2020). Structure-based design of prefusion-stabilized SARS-CoV-2 spikes. Science *369*, 1501–1505.

37. Rogers, T.F., Zhao, F., Huang, D., Beutler, N., Burns, A., He, W.-T., Limbo, O., Smith, C., Song, G., Woehl, J., et al. (2020). Isolation of potent SARS-CoV-2 neutralizing antibodies and protection from disease in a small animal model. Science *369*, 956–963.

38. Wibmer, C.K., Ayres, F., Hermanus, T., Madzivhandila, M., Kgagudi, P., Oosthuysen, B., Lambson, B.E., de Oliveira, T., Vermeulen, M., van der Berg, K., et al. (2021). SARS-CoV-2 501Y.V2 escapes neutralization by South African COVID-19 donor plasma. Nat. Med. *27*, 622–625.

39. Spencer, A.J., Morris, S., Ulaszewska, M., Powers, C., Kailath, R., Bissett, C., Truby, A., Thakur, N., Newman, J., Allen, E.R., et al. (2021). The ChAdOx1 vectored vaccine, AZD2816, induces strong immunogenicity against SARS-CoV-2 B.1.351 and other variants of concern in preclinical studies. bioRxiv. https://doi.org/10.1101/2021.06.08.447308.

40. Yuan, M., Huang, D., Lee, C.D., Wu, N.C., Jackson, A.M., Zhu, X., Liu, H., Peng, L., van Gils, M.J., Sanders, R.W., et al. (2021). Structural and functional ramifications of antigenic drift in recent SARS-CoV-2 variants. Science *373*, 818–823.

41. Becker, M.M., Graham, R.L., Donaldson, E.F., Rockx, B., Sims, A.C., Sheahan, T., Pickles, R.J., Corti, D., Johnston, R.E., Baric, R.S., and Denison, M.R. (2008). Synthetic recombinant bat SARS-like coronavirus is infectious in cultured cells and in mice. Proc. Natl. Acad. Sci. USA *105*, 19944–19949.

42. Corman, V.M., Landt, O., Kaiser, M., Molenkamp, R., Meijer, A., Chu, D.K., Bleicker, T., Brünink, S., Schneider, J., Schmidt, M.L., et al. (2020). Detection of 2019 novel coronavirus (2019-nCoV) by real-time RT-PCR. Euro Surveill. *25*, 2000045.

43. Downing, L., Sawarynski, K.E., Li, J., McGonagle, M., Sims, M.D., and Marples, B. (2010). A simple quantitative method for assessing pulmonary damage after x irradiation. Radiat. Res. *173*, 536–544.

STAR★METHODS

KEY RESOURCES TABLE

REAGENT or RESOURCE	SOURCE	IDENTIFIER
**Bacterial and virus strains**		
Wild-type SARS-CoV-2 isolate	Jonas Klingström	N/A
501Y.V2 isolate	Alex Sigal	Cele et al.[34]
**Biological samples**		
Plasma from NHPs	This study	N/A
**Chemicals, peptides, and recombinant proteins**		
Recombinant SARS-CoV-2 WT Spike	This study	N/A
Recombinant SARS-CoV-2 501Y.V2 RBD	This study	N/A
Polyethylenimine	Sigma-Aldrich	Cat# 764604
Matrix-M	Novavax AB	N/A
Lipofectamine 3000	Invitrogen	Cat# L3000075
Bright-Glo Luciferase Assay System	Promega	Cat# E2620
TRIzol Reagent	Thermo Fisher Scientific	Cat# 15596026
GlycoBlue Coprecipitant	Thermo Fisher Scientific	Cat# AM9515
SuperScript III One-Step RT-PCR System with Platinum Taq DNA Polymerase	Thermo Fisher Scientific	Cat# 12574018
mMESSAGE mMACHINE T7 Transcription Kit	Thermo Fisher Scientific	Cat# AM1344
GIBCO FreeStyle MAX Reagent	Thermo Fisher Scientific	Cat# 16447100
Gibson Assembly Mastermix	New England Biolabs	Cat# E2611S
**Deposited data**		
SARS-CoV-2 lineage metadata	GISAID	https://www.gisaid.org
**Experimental models: cell lines**		
Human: GIBCO FreeStyle 293-F cells	Thermo Fisher Scientific	Cat# R79009
Human: HEK293T-ACE2	Hanke et al.[35]	N/A
Human: Calu-3	Jonas Klingström	N/A
African Green Monkey: Vero E6 cell	ATCC	Cat# CRL-1586; RRID: CVCL_0574
**Experimental models: organisms/strains**		
NHP: Macaca mulatta	N/A	N/A
Mouse: K18-hACE2	Jackson Laboratory, McCray et al.[24]	Cat# 034860; RRID: MSR_JAX:034860
**Oligonucleotides**		
gBlocks Gene Fragments	Integrated DNA Technologies	N/A
**Recombinant DNA**		
SARS-CoV-2 Spike ectodomain plasmid (expression)	Hsieh et al.[36]	Addgene: 154754; RRID: Addgene_154754
SARS-CoV-2 B.1.351/501Y.V2 RBD plasmid	This study	N/A
SARS-CoV-2 WT Spike plasmid	James Voss	Rogers et al.[37]
SARS-CoV-2 B.1.1.7 Spike plasmid	David Nemazee	Addgene: 170451; RRID: Addgene_170451
SARS-CoV-2 B.1.351 Spike plasmid	Penny Moore	Wibmer et al.[38]
SARS-CoV-2 P.1 Spike plasmid	David Nemazee	Addgene: 170450; RRID: Addgene_170450
SARS-CoV-2 B.1.617.2 Spike plasmid	G2P-UK National Virology consortium	Spencer et al.[39]
SARS-CoV-1 Spike plasmid	James Voss	Rogers et al.[37]
Lentiviral backbone: pCMV delta R8.2	Bob Weinberg	Addgene: 8455; RRID: Addgene_8455

(Continued on next page)

Continued

REAGENT or RESOURCE	SOURCE	IDENTIFIER
Luciferase transfer plasmid	James Voss	Rogers et al.[37]
**Software and algorithms**		
GraphPad Prism v9.0.0	GraphPad Software Inc.	https://www.graphpad.com/scientific-software/prism/; RRID: SCR_002798
Julia v1.6	The Julia Programming Language	https://julialang.org/
Non-linear Multinomial Regression for VOC frequency estimation	This study	https://github.com/MurrellGroup/VOCfreq; https://doi.org/10.5281/zenodo.5562800
**Other**		
HiLoad® 16/600 Superdex® 200 pg	Cytivia	Cat# 28-9893-35
His-Pur Ni-NTA resin	Thermo Fisher Scientific	Cat# 88222

RESOURCE AVAILABILITY

Lead contact

Further information and requests for resources or reagents should be directed to and will be fulfilled by the Lead Contact, Ben Murrell (benjamin.murrell@ki.se).

Materials availability

All unique/stable reagents generated in this study are available from the Lead Contact with a completed Materials Transfer Agreement.

Data and code availability

Original data reported in this paper will be shared by the Lead Contact upon request.

All original code has been deposited at https://github.com/MurrellGroup/VOCfreq and is publicly available as of the date of publication. DOIs are listed in the Key resources table.

Any additional information required to reanalyze the data reported in this work paper is available from the Lead Contact upon request.

EXPERIMENTAL MODEL AND SUBJECT DETAILS

Ethics statement

The animal work was conducted with the approval of Stockholms Jordbruksverket (10513-2020, 18427-2019 and 10895-2020). All animal procedures were performed according to approved guidelines.

Animal models

Rhesus macaques

Three female rhesus macaques (*Macaca mulatta*) of Chinese origin, 5-6 years old, were housed at the Astrid Fagraeus Laboratory at Karolinska Institutet. Housing and care procedures complied with the provisions and general guidelines of the Swedish Board of Agriculture. The facility has been assigned an Animal Welfare Assurance number by the Office of Laboratory Animal Welfare (OLAW) at the National Institutes of Health (NIH). The macaques were housed in groups in enriched 14 m^3 cages. They were habituated to the housing conditions for more than six weeks before the start of the experiment and subjected to positive reinforcement training in order to reduce the stress associated with experimental procedures. The macaques were weighed at each sampling. All animals were confirmed negative for simian immunodeficiency virus, simian T cell lymphotropic virus, simian retrovirus type D and simian herpes B virus.

K18-hACE2 mice

Mice transgenic for human ACE2 under control of the cytokeratin 18 (K18) promoter[24] were obtained from the Jackson Laboratory. Mice were maintained as a hemizygous line, with hACE2 transgene presence confirmed using Sanger sequencing as per the Jackson Laboratory protocol. All mice were 12-21 weeks old at the start of the study, and experiments were conducted in BSL3 facilities at the Comparative Medicine department (KM-F) at Karolinska Institutet. Mice (24 male, 20 female) were housed in individually ventilated cages, had access to food and water *ad libitum*, and cage enrichment included shredded cardboard and paper rolls. Cage and water changes were performed on a weekly basis and general monitoring of all mice was performed daily by technical staff.

Cell lines

HEK293T and HEK293T-hACE2 (Human, female) cells were cultured in a humidified 37°C incubator (5% CO_2) in Dulbecco's Modified Eagle Medium (GIBCO) supplemented with 10% Fetal Bovine Serum and 1% Penicillin/Streptomycin, and were passaged when nearing confluency using 1X Trypsin-EDTA.

Calu-3 cells (Human, male), a lung-derived adenocarcinoma cell line, were obtained from Jonas Klingstrom (Karolinska Institutet). Calu-3 cells were maintained in Dulbecco's Modified Eagle Medium supplemented with Ham's F-12 (Thermo Fisher Scientific), 2% Fetal Bovine Serum and 1% Penicillin/Streptomycin in a humidified 37°C incubator (5% CO_2) and were passaged using 0.5X Trypsin-EDTA.

Vero E6 cells (ATCC-CRL-1586, African Green Monkey) were maintained in DMEM (GIBCO) supplemented with 2% fetal calf serum and 1% penicillin-streptomycin in a humidified incubator with 5% CO_2 at 37°C.

METHOD DETAILS

Immunizations

501Y.V2 RBD (encoding amino acid mutations K417N, E484K, and N501Y, and a C-terminal His-tag) was synthesized (Integrated DNA Technologies), and cloned into a mammalian expression vector (pcDNA3.1), using a Gibson Assembly Mastermix (New England Biolabs). Spike ectodomain (prefusion stabilized with 6 prolines[36]) and RBD were produced by the transient transfection of Freestyle 293-F cells using FreeStyle MAX reagent (Thermo Fisher) or polyethylenimine (PEI), respectively. The HIS-tagged Spike ectodomain and RBD were purified from filtered supernatant using nickel IMAC resin (HisPur Ni-NTA, Thermo Fisher Scientific) followed by size-exclusion chromatography on a Superdex 200 (Cytiva) in PBS. On the day of immunization, indicated doses were mixed with 50 μg Matrix-M adjuvant (Novavax AB, Uppsala, Sweden) in a final inoculation volume of 800 μl.

Macaques were immunized intramuscularly (i.m.) with half of each dose administered in each quadricep. All immunizations and blood samplings were performed under sedation with 10-15 mg/kg ketamine (Ketaminol, Intervet, Sweden) administered i.m.. Blood plasma was isolated by centrifugation, and heat inactivated at 56°C for 60 min.

Pseudotyped virus neutralization assays

All plasma and serum samples were heat inactivated at 56°C for 60 min. Pseudotyped lentiviruses displaying spikes (with C-terminal truncations) from the SARS-CoV-2 pandemic founder variant (Wu-Hu-1)[37] or from variants of concern[38–40] and packaging a firefly luciferase reporter gene were generated by the co-transfection of HEK293T cells using Lipofectamine 3000 (Invitrogen) per the manufacturer's protocols. Media was changed 12-16 h after transfection, and pseudotyped viruses were harvested at 48- and 72-h post-transfection, clarified by centrifugation, and stored at −80°C until use. Pseudotyped viruses sufficient to generate ~50,000 relative light units (RLUs) were incubated with serial dilutions of plasma for 60 min at 37°C in a 96-well plate, and then ~15,000 HEK293T-hACE2 cells were added to each well. Plates were incubated at 37°C for 48 h, and luminescence was then measured using Bright-Glo (Promega) per the manufacturer's protocol, on a GM-2000 luminometer (Promega).

Virus production and quantification

Viral isolates expanded from clinical samples of 'wild-type' and 501Y.V2[34] were kind gifts from Jonas Klingström (Karolinska Institutet) and Alex Sigal (The African Health Research Institute) respectively. Isolates were propagated in Calu-3 cells for 72 h and harvested from the supernatant to generate replication-competent SARS-CoV-2 stocks for animal challenge. All virus challenge stocks harbored no cell-culture adaptation mutations detectable by Sanger sequencing of the full-length spike gene (see Figure S4). Viral titers were quantified by a plaque-forming assay in Vero E6 cells, as previously described[41].

Viral RNA was quantified from clarified viral supernatant in TRIzol (ThermoFisher Scientific) and extracted using the manufacturer's protocol but with the following modifications: Total RNA was precipitated with isopropanol in the presence of Glycoblue (Thermo Fisher Scientific) coprecipitant for 45 min at −20°C, and RNA pellets were resuspended in warm RNase-free water.

RT-qPCR reactions were performed using the Superscript III one step RT-qPCR system with Platinum Taq Polymerase (Invitrogen) with 400 nM of each primer and 200 nM of probe. Primers and probes for the CoV-E gene target were as previously described[42]. Thermal cycling consisted of RT at 55°C for 10 min, denaturation at 95°C for 3 min, and 45 cycles of 95°C for 15 s and 58°C for 30 s. Reactions were carried out using a CFX96 Connect Real-Time PCR Detection System (Bio-Rad) following manufacturer instructions. To generate standard curves, a synthetic DNA template gBlock (Integrated DNA Technologies) was transcribed using the mMessage mMachine T7 Transcription Kit (Invitrogen) and serially diluted. For each viral stock, RT-qPCR was run on a 10-fold dilution series ranging from 1:10 to 1:10,000 to ensure linearity of the assay and avoid saturation at high copy numbers.

K18-hACE2 mice challenge

One day prior to challenge, K18-hACE2 mice were passively immunized with 200 μL of macaque plasma, administered intraperitoneally (i.p.) under isoflurane sedation. Each plasma sample was administered to four mice (two to be challenged with wild-type virus, and two to be challenged with 501Y.V2). The following day, mice were bled from the tail vein (to obtain serum for the characterization of neutralizing antibody titers), moved to a BSL3 facility and challenged intranasally with a standardized dose (2.4×10^6 RNA copies) of either 'wild-type' or 501Y.V2 virus stock in a total challenge volume of 40 μL in PBS. All challenges were performed under

light isoflurane sedation. Weight and general body condition was monitored daily until weight loss was evident, after which mice were monitored twice daily. Throughout the experiment, weight loss, changes in general health, breathing, body movement and posture, piloerection and eye health were monitored. Mice were euthanized when they experienced weight loss of at least 20% of their starting body weight, or when movement was greatly impaired and/or they experienced difficulty breathing that was considered to reach a severity level of 0.5 on Karolinska Institutet's veterinary plan for monitoring animal health.

QUANTIFICATION AND STATISTICAL ANALYSIS

Non-linear Multinomial Regression for VOC frequency estimation

For plotting maps in Figure 1, SARS-CoV-2 lineage metadata was obtained from GISAID (gisaid.org - 2021-10-09 metadata release), comprising 4,197,215 genomes. Pango lineages without WHO labels were collapsed up the Pango lineage tree until a minimum of 50,000 observations for each lineage was obtained, and daily counts of genomes were aggregated at the country level. We used a multinomial regression to infer the frequencies at three selected dates, weighting the observations around the target dates using a radial basis function. Note: the lineage frequencies vary over time, but the relative advantage of one variant over another is assumed by the model to be locally constant around the target date. Inference was performed with the GLMNet.jl Julia package, using an alpha = 0.5, and maps were plotted with the Cartopy python package (https://github.com/SciTools/cartopy). Jupyter notebooks implementing this are available at https://github.com/MurrellGroup/VOCfreq.

ID_{50} titers

Neutralizing antibody ID_{50} titers were calculated in Prism 9 (GraphPad Software) by fitting a four-parameter logistic curve bounded between 0 and 100, and interpolating the reciprocal plasma/serum dilution where RLUs were reduced by 50% relative to control wells (N = 8) in the absence of plasma.

Histology

Pulmonary pathology in blinded H&E-stained lung sections (N = 89 slides in total, with a mean of 3.3 slides per mouse (range: 2-5) was scored according to a four-point scale, as described previously[43]. Briefly, normal architecture with thin alveolar septa and distinct bronchioles is scored as 0. Sections with thickened alveolar septa and reduced alveolar space are scored as 1. Thickening of the alveolar septa with reduced alveolar space and invasion of inflammatory cells is scored as 2. Sections displaying widespread loss of tissue architecture are scored as 3. The score for each animal represents the mean of the pathology scores for all sections from that animal. Representative images from each group are shown in Figure S5.

Statistical Analysis

All statistical analyses were performed in Prism 9 (Graphpad Software) unless otherwise stated. Differences in day 6 weights between groups were assessed using Mann-Whitney tests. Reduction in pathology scores was assessed with a one-tailed Mann-Whitney test. Depicted symbols summarize the following: ns = $p > 0.05$ (not significant), * $p \leq 0.05$, ** $p \leq 0.01$, *** $p \leq 0.001$. The association between neutralizing antibody titers and weight loss was assessed with a Spearman's rank correlation.

Cell Reports Medicine, Volume *2*

Supplemental information

Beta RBD boost broadens antibody-mediated protection against SARS-CoV-2 variants in animal models

Daniel J. Sheward, Marco Mandolesi, Egon Urgard, Changil Kim, Leo Hanke, Laura Perez Vidakovics, Alec Pankow, Natalie L. Smith, Xaquin Castro Dopico, Gerald M. McInerney, Jonathan M. Coquet, Gunilla B. Karlsson Hedestam, and Ben Murrell

Supplementary Figures

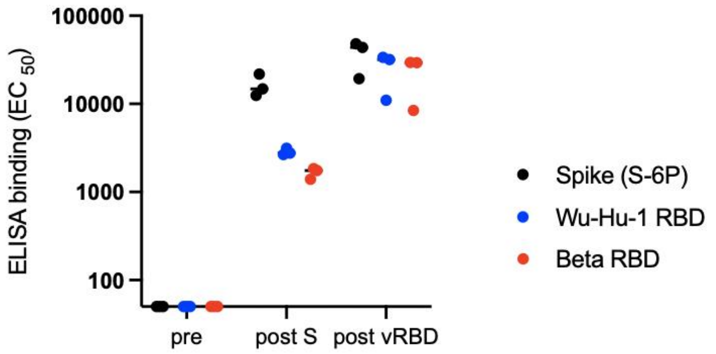

Supplementary figure 1. ELISA spike and RBD binding titers. Binding antibody responses by ELISA are shown for samples prior to immunization (pre) and 2 weeks following the second spike immunization (post S) or variant RBD boost (post vRBD). Binding was assessed against Spike ectodomain with 6 Proline mutations stabilizing the pre-fusion conformation (S-6P, black), as well as against RBD matching Wu-Hu-1 (blue) and the Beta VOC (red). Related to Figure 2.

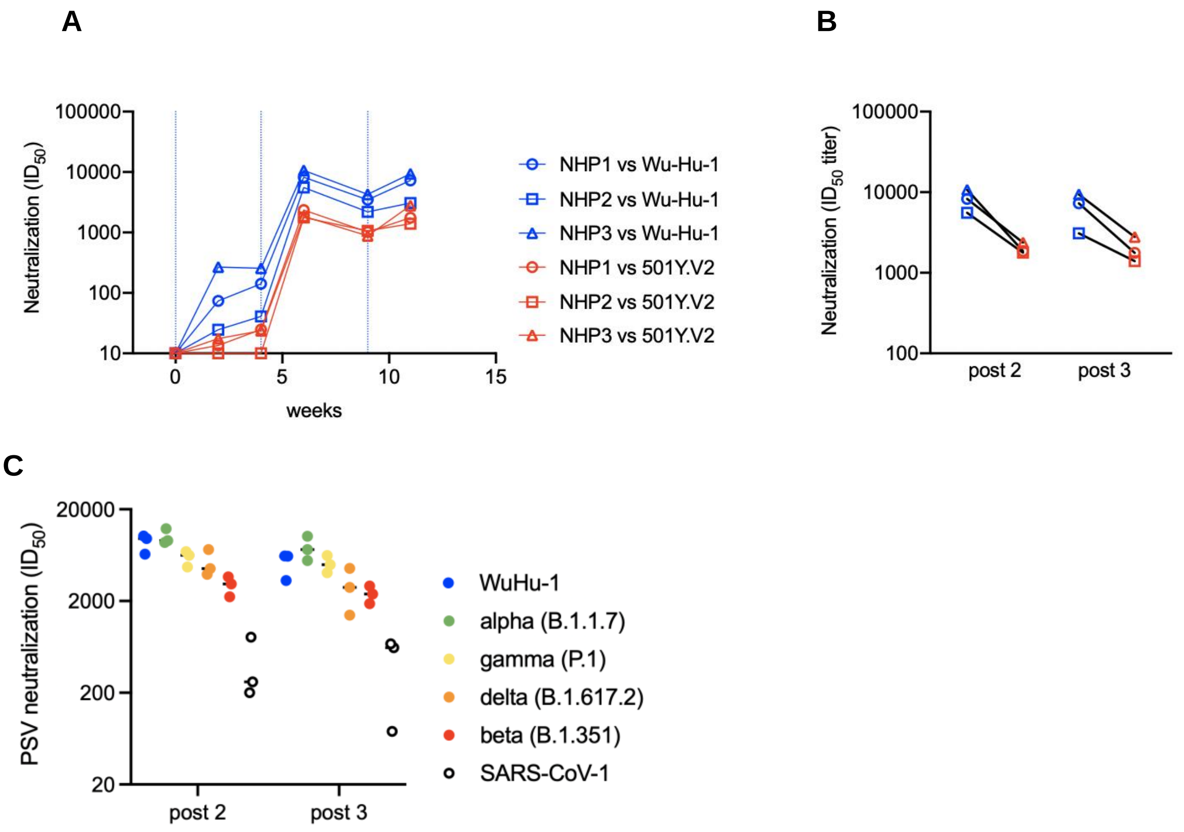

Supplementary figure 2. (**a**) Longitudinal neutralizing antibody responses against Wu-Hu-1 (blue) and 501Y.V2 (red) for plasma samples from [21], where three rhesus macaques (NHP1-NHP3) were immunized with three doses of Wu-Hu-1 spike (100 µg) in Matrix-M™ adjuvant. Vertical blue lines indicate the timing of immunizations (at 0, 4, and 9 weeks). (**b-c**) Comparison of the neutralizing antibody titers at 6 weeks (post 2) and 11 weeks (post 3) illustrating that reduced titers to beta (red) and other VoCs (**c**) compared to Wu-Hu-1 (blue) persisted after a third homotypic spike boost. Related to Figure 2.

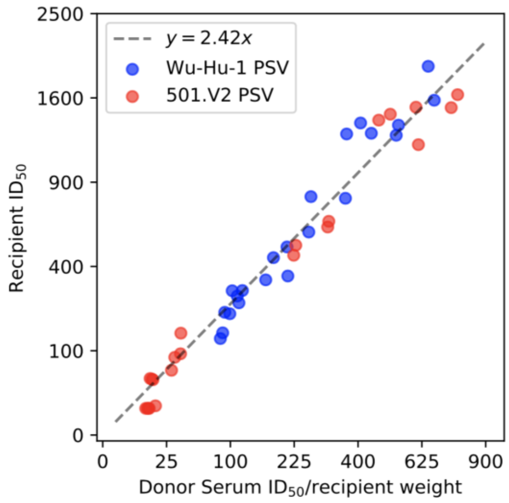

Supplementary figure 3: Relationship between donor and recipient titers following passive immunization. Mice were passively immunized with 200 ul of plasma, resulting in titers approximately 10-fold lower than in the donor. Correcting for weight, Spearman's r = 0.98 (after a variance-stabilizing square-root transform). Related to Figure 3.

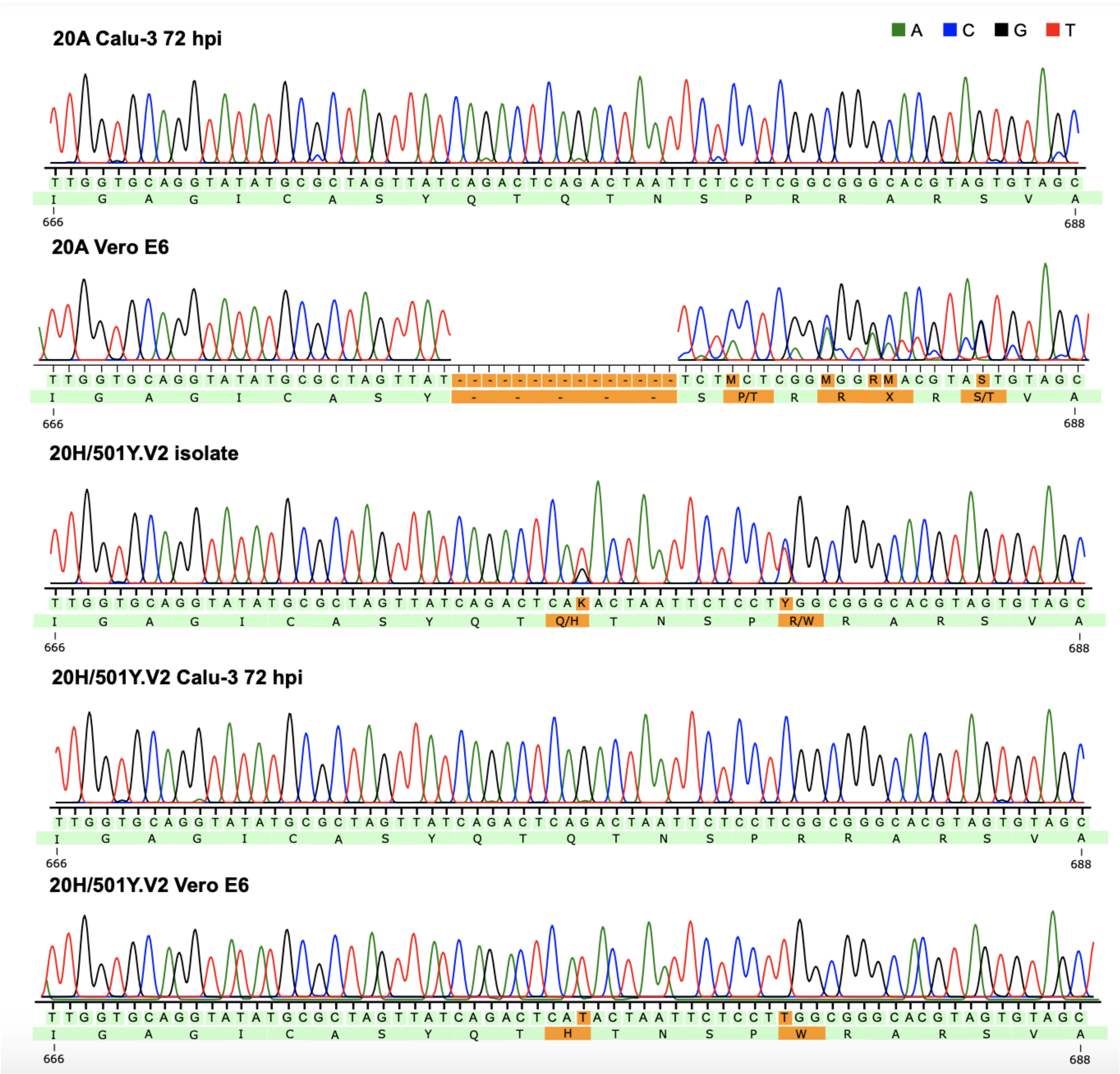

Supplementary figure 4. Expansion of SARS-CoV-2 isolates in Vero E6 but not Calu-3 cells rapidly selected for mutations and deletions proximal to the furin cleavage site. Challenge stocks used in this study were produced in Calu-3 cells, and confirmed by Sanger sequencing to harbour no high frequency cell culture adaptation mutations in spike. Electropherograms spanning the furin cleavage site from sanger sequencing of amplified viral RNA are shown for virus cultured in Vero E6 or Calu-3 cells demonstrating the rapid loss of the furin recognition sequence upon culture in Vero E6 cells but not Calu-3 cells. Received stock of 501Y.V2 ("20H/501Y.V2 isolate") had a mixture of intact/knocked-out furin site. Related to Figure 3.

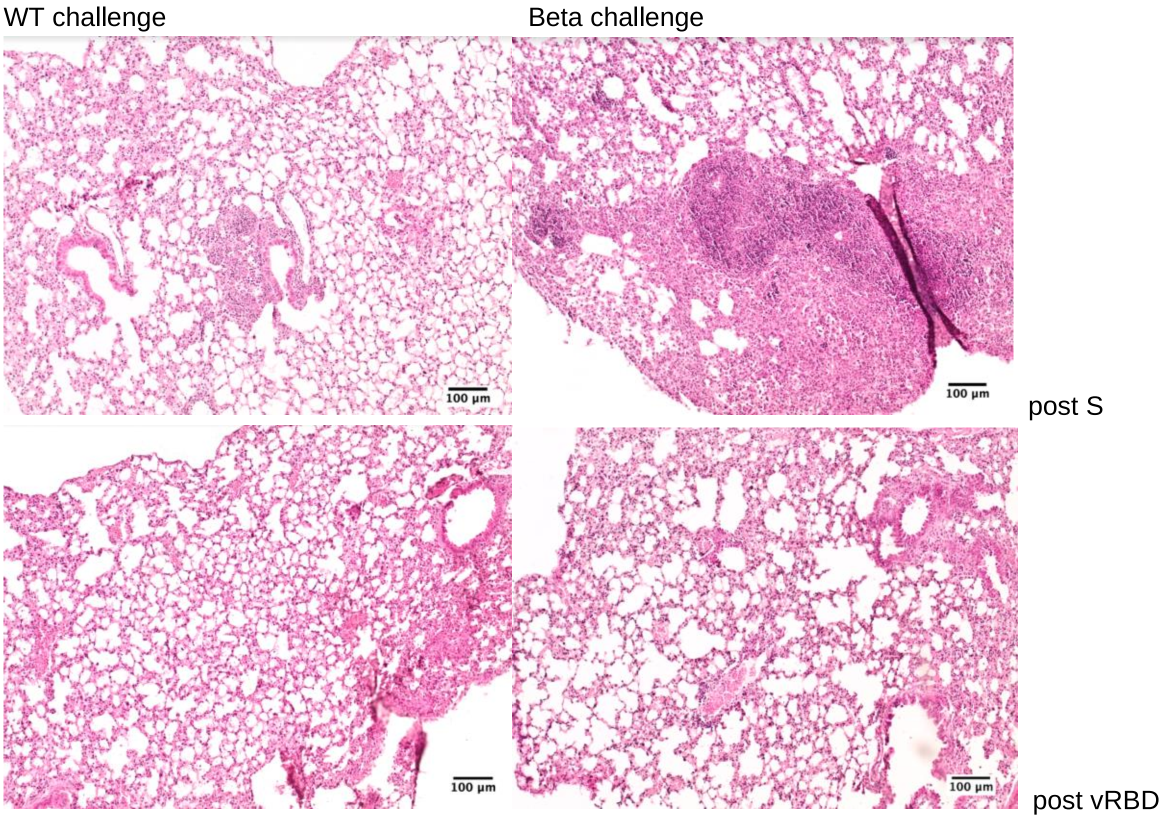

Supplementary figure 5. Lung histopathology in surviving mice. Representative images of Hematoxylin and Eosin stained lung sections from surviving mice at day 14, illustrating typical histopathology in each group. Scale bar represents 100 µm. Related to Figure 3f.
